# A robust dual reporter system to visualize and quantify gene expression mediated by transcription activator-like effectors

**DOI:** 10.1186/1480-9222-14-8

**Published:** 2012-08-08

**Authors:** Claudia Uhde-Stone, Joseph Huang, Biao Lu

**Affiliations:** 1 Departments of Biological Sciences, California State University, East Bay, Hayward CA 94542, USA; 2 System Biosciences (SBI), 265 North Whisman Road, Mountain View CA 94043, USA

**Keywords:** Dual reporter, Gene editing, Transcription activator-like effector, Green fluorescent protein, Firefly luciferase

## Abstract

**Background:**

Transcription activator-like effectors (TALEs) are a class of naturally occurring transcription effectors that recognize specific DNA sequences and modulate gene expression. The modularity of TALEs DNA binding domain enables sequence-specific perturbation and offers broad applications in genetic and epigenetic studies. Although the efficient construction of TALEs has been established, robust functional tools to assess their functions remain lacking.

**Results:**

We established a dual reporter system that was specifically designed for real-time monitoring and quantifying gene expression mediated by TALEs. We validated both sensitivity and specificity of this dual-reporter system in mammalian cells, and demonstrated that this dual reporter system is robust and potentially amenable to high throughput (HTP) applications.

**Conclusion:**

We have designed, constructed and validated a novel dual reporter system for assessing TALE mediated gene regulations. This system offers a robust and easy-to- use tool for real-time monitoring and quantifying gene expression in mammalian cells.

## Background

Zinc-finger nucleases (ZFNs) and meganucleases have been well-established as tools to edit specific sites in complex genomes [1,2]. However, the DNA-binding domain of zinc-finger nucleases are challenging to design and require experimental optimization, and the target sequences of meganucleases are limited. Transciption activator-like effector (TALE) technology has recently emerged as an alternative robust and efficient genome editing tool [3,4]. Natural TALEs are transcription factors used by plant-pathogenic bacteria in the genus Xanthomonas. The native function of TALEs is to modulate host gene expression by binding to specific sequences in host gene promoters and activate transcription [5,6]. The DNA binding domain of TALEs consist of a central domain of 33–35 amino acid repeats arranged in tandem, followed by a single truncated repeat of 20 amino acids. The tandem repeats are nearly identical, except for two variable amino acids in position 12 and 13, referred to as “repeat-variable di-residue” (RVD), with the four most common RVDs each specifying binding of one of the four DNA bases [7,8]. The modular nature of the TALE DNA-binding domain and the straightforward sequence relationships enables efficient customization of TAL effector repeat domains. The simplicity of TALE design and construction to target nearly any DNA sequence within the genome has been an important advantage of the technology [9,10].

TALEs with customized DNA-binding domains can be fused to the catalytic domain of the FokI nuclease to create targeted DNA double-strand breaks in vivo for genome editing [11-15]. TAL effector nucleases (TALENs) function in pairs, as FokI cleaves DNA upon dimerization. Double-strand breaks will be repaired in most cells by non-homologous end joining, which frequently results in small deletions or insertions, thus leading to potential gene disruption. Alternatively, homologous recombination in presence of a donor sequence can be harnessed for gene insertion or replacement [4]. Recently, engineered TALENs have been shown as a robust gene editing tool in a number of species [4,16-20]. Similarly, sequence-specific TALEs for modulating eukaryotic gene activation or repression have also been reported [21,22]. With the availability of efficient assembly of TALEs for use in mammalian cells, attention starts to shift to develop functional monitoring tools. Although a single reporter system using either fluorescent protein or luciferase has been reported, neither system is commercially available or amenable to HTP applications [23]. Here we demonstrated the design, construction and validation of a novel dual-reporter system for the real-time monitoring and quantification of gene expression mediated by TALEs. With the simplicity and robustness of this dual reporter system, this novel tool offers a solution for functional assessment of gene regulation mediated by TALEs with high sensitivity and specificity.

## Results and Discussion

### Features of the dual reporter for TALEs

**Figure 1:**
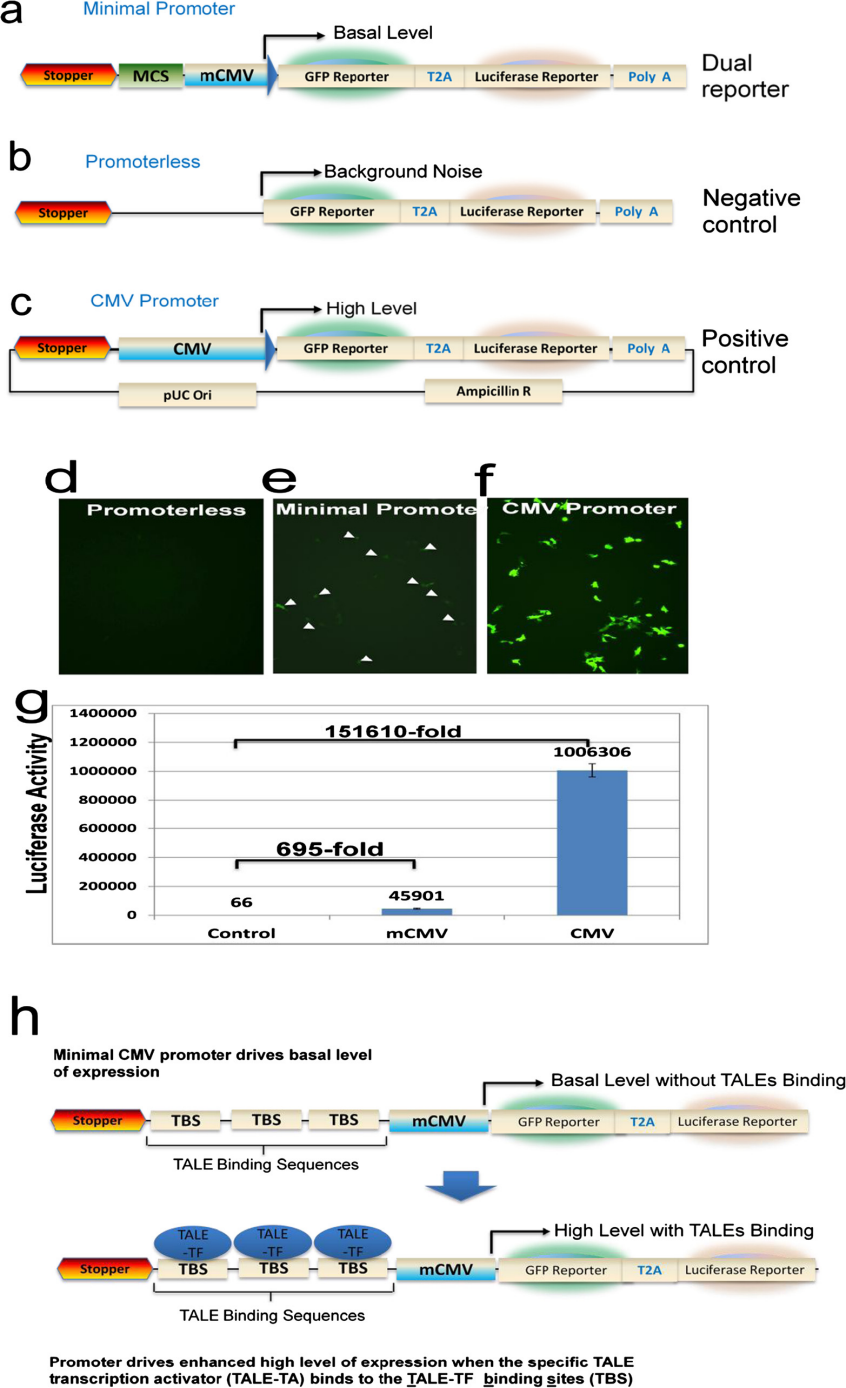
**Design and Construction of the dual reporters and their functional characterization. **(**a**) Schematic representation of core features of the dual reporter system, depicting the two reporter genes GFP and luciferase separated by T2A (self-cleavage peptide), under the control of a minimal CMV promoter (mCMV). The multiple cloning sites (MCS) were incorporated to facilitate the insertion of TALE binding sequences. (**b**) Schematic representation of the promoterless dual reporter system serving as a negative control. (**c**) Schematic representation of the dual reporters under the control of a full CMV promoter serving as a positive control. HEK293 cells transfected with the negative control (promoterless) displayed weak GFP expression (**d**), while the minimal CMV promoter resulted in low to moderate GFP expression (**e**); the positive control (full CMV promoter) showed strong GPP expression (**f**). (**g**) Luciferase activities of each reporter, displayed in arbitrary units. Error bars indicate SD; n = 3. (**h**) Flow chart showing how to use the dual reporter to monitor TALE action. Specific TALE reporters can be constructed by inserting the TALE binding sequence (TBS) into the MCS. These customized reporters can then be used to trace TALE action in mammalian cells by co-transfection with the corresponding TALE.

The dual reporter for TALEs was designed to make use of the unique property of two reporters: green fluorescent protein (GFP) and firefly luciferase. While GFP by itself offers a visual, real-time monitoring of gene activation, the quantification is time-consuming and requires the use of expensive analysis machine such as flow cytometry. To overcome this limitation, we designed a dual reporter, which combines GFP with a quantifiable luciferase (Figure 1a). Because luciferase reporter can provide a cost-effective way of quantification, together this dual reporter system offers a powerful tool superior to any single reporter system.

To monitor TALE-induced gene expression, a minimal promoter is required to turn on the transcription of down-stream reporters. We chose the minimal CMV promoter (mCMV), which has been demonstrated to function in various mammalian cells. To minimize the non-specific influence of transcription, cares were taken to mutate the potential transcription binding sites within the backbone. In addition, a transcription stopper sequence was placed before the TALE binding sites, directly upstream of a multiple cloning site (MCS) (Figure 1a). To evaluate the background activities, a promoterless reporter was constructed as negative control (Figure 1b). Similarly, a full-length CMV promoter reporter was constructed as positive control to validate experimental parameters such as transfection conditions and efficiencies (Figure 1c).

We next determined the function of these reporters in mammalian cells. As expected, transfection of human embryonic kidney cells (HEK293) with promoterless reporter showed no GFP-positive cells and was used to establish background-level luciferase activity (Figure 1d). Under the same experimental conditions, a few GFP-positive cells were detected for the dual reporter with mCMV promoter (Figure 1e). In contrast, more than 85% cells showed GFP positive for the positive control reporter (Figure 1f). A low to moderate level (695-fold increase vs. promoterless control) of luciferase activity was detected for the minimal promoter, while a very robust level (>150000-fold increase vs. promoterless controls) was observed for the positive control reporter (Figure 1g). These results demonstrated the desired functions of our dual reporter system. Two key features are worthy to note: first, the dual reporter has very low background noise and potentially very high signal levels in mammalian cells (HEK293 cells, Figure 1). Secondly, the low to moderate basal activity of the dual reporter offers a potential capability to monitor both an increase or decrease in gene expression (unpublished data).

To facilitate monitoring the transcription activity of TALE-TF (transcription factor), multiple cloning sites were readily placed at the immediate upstream of the mCMV. In this way, TALE-TF binding sites will be in optimal position for transcriptional activation (within 200 bp upstream of transcription start sites). Of note ~3-5 binding sites are normally engineered into reporter construct to increase the sensitivity of reporters (Figure 1h).

### Specificity of dual reporters for TALE-TF

**Figure 2:**
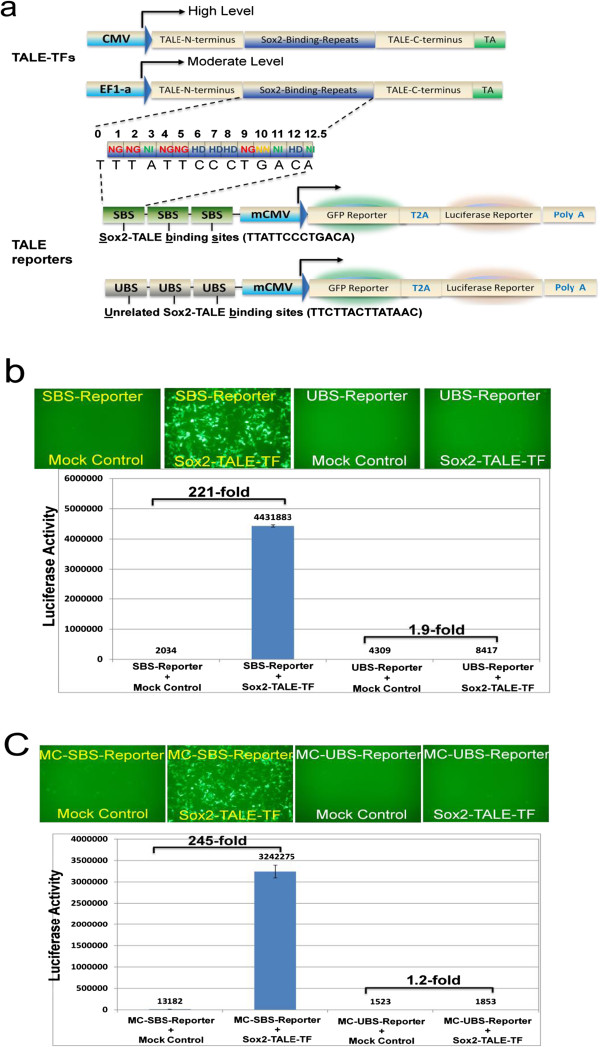
**Specific activation of dual reporters by TALEs. **(**a**) **Upper two panels:** TALE-TF designed to activate the Sox2 gene were placed under the control of either CMV or EF1-a promoter. The target sites were selected from the 200-bp proximal promoter region of human Sox2 as indicated by Sox2-TALE binding sites (SBS). **Lower two panels:** the corresponding customized dual reporter was constructed by inserting 3xSBS. A control reporter harboring 3xUBS was set as a negative control. (**b**) **Upper panel:** Images of TALE-induced GFP reporter expression in trasnfected HEK293 cells. **Lower panel**: Levels of luciferase activities in transfected HEK293 cells, as determined by quantitative luciferase assay, in comparison to mock controls. Mock control cells received the same amount of plasmid DNA, but the Sox2 TALE-TF plasmid was replaced by empty vector. Data are expressed as mean ± SD, n = 3.

We next investigated the specificity of dual reporters in the context of monitoring TALE-TF. The programmable nature of TALEs allows for a virtually arbitrary selection of any target DNA-binding site. We chose the Sox2-TALE-TF, as this TALE has been previously demonstrated to be able to activate both the endogenous Sox2 gene and a reporter [24]. As shown in Figure 2, we constructed two Sox2-TALE-TF expression vectors: one with a CMV promoter to achieve high expression and the other with an EF1a promoter to achieve moderate expression (Figure 2a). The Sox2-TALE-TF binding sequences were deduced from the Sox2 effector binding domain, and used for construction of its dual reporter (Figure 2a). Specifically, a double-stranded DNA fragment containing three binding sites were synthesized, PCR-amplified and subsequently cloned into the MCS. To avoid potential interference of DNA binding to the tandem binding sites, each target sites were inserted with an arbitrary spacer (5-bp). A similar reporter with unrelated repeat sequences was also built as negative control (Figure 2a).

To evaluate the specificity of the above reporters, a co-transfection experiment was conducted in HEK293 cells. As shown in Figure 2b (the upper 2^nd^ panel), when the Sox2 binding site reporter was co-transfected with the Sox2-TALE-TF, >85% HEK293 cells were GFP positive. In contrast, barely any GFP positive cells showed up in the mock transfection controls using empty vector DNA lacking the Sox2-TALE-TF (Figure 2b, the upper 1^st^ panel). Correspondingly, a 221-fold increase in luciferase activity was observed in co-transfected cells, while the mock control displayed only low activity. Because the only difference of these two constructs are the TALE-binding sites, these data strongly support that the dual reporter system is both a sensitive and a specific method to validate TALE activity.

To further confirm that this dual reporter configuration is useful in validating TALEs function, a new set of dual reporters were built with a different plasmid backbone, namely the minicircle plasmid DNA. To demonstrate the specificity of the dual reporter in these new vector backbones, the same co-transfection experiments were conducted. As shown in Figure 2c, Sox2-TALE-TF specifically activated reporter genes to a similar extend, with a drastic increase in the numbers of GFP-positive cells, and a 245-fold increase in luciferase activity. These results further validated that the dual reporter is useful for specifically monitoring gene activation mediated by TALEs, and indicate that the core configuration of the dual reporter can be applied to a broad range of plasmid backbones.

### Sensitivity of the dual reporter system

**Figure 3:**
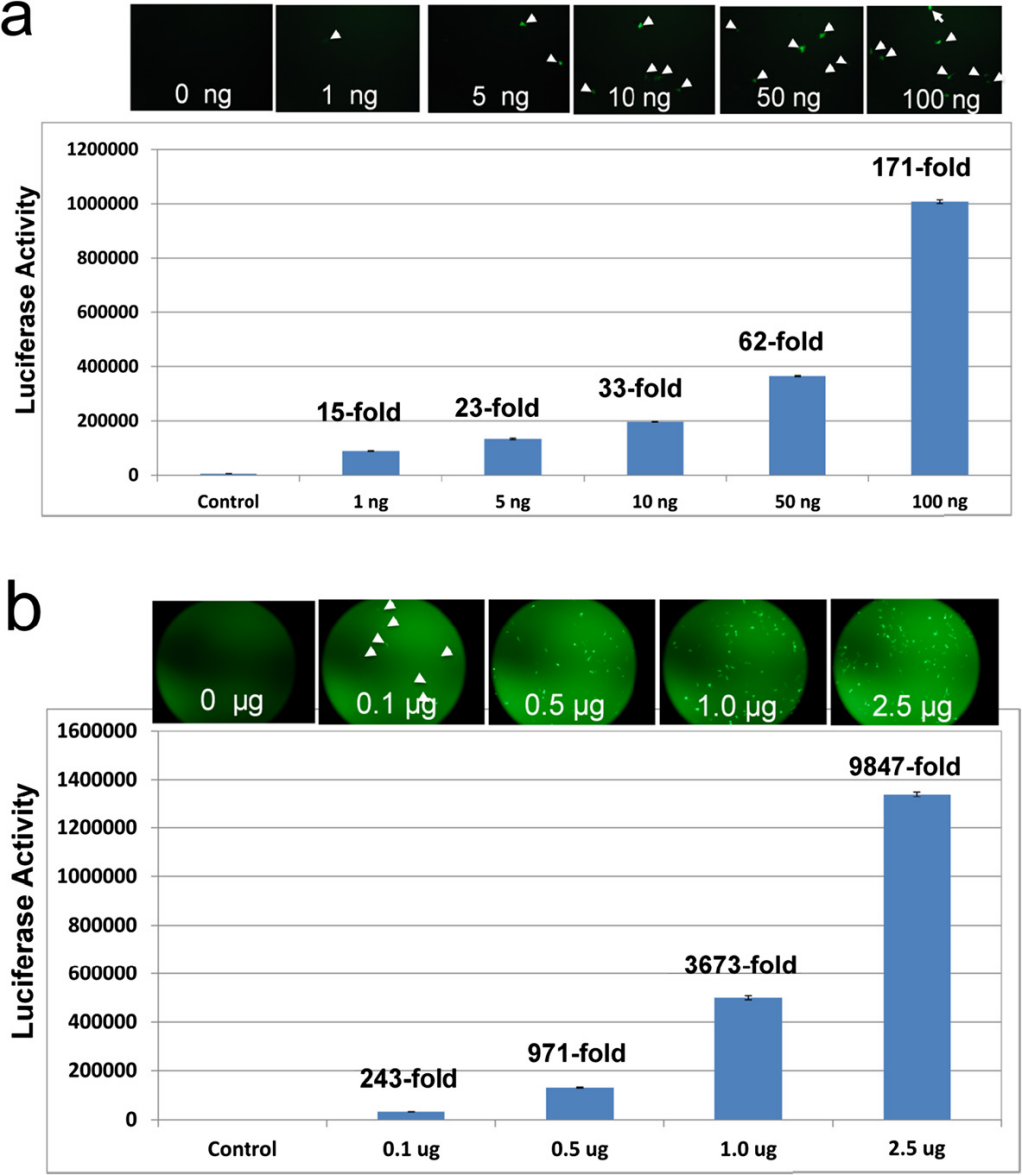
**The robustness of dual reporters. **(**a and b, upper panels**)**:** Images of TALE-induced GFP reporter expression in HEK293 cells. (**a**) Low-range of Sox2-TALE-TF input, images taken at 20x magnification (**b**) High-dose range of Sox2-TALE-TF input, images taken at 10x magnification. (**a and b, lower panels**)**:** Levels of luciferase activities expressed as mean ± SD, n = 3 at low-dose (**a**) and high-dose (**b**) range of Sox2-TALE-TF input. The fold induction of the reporter was calculated as relative activity of each dosage compared to the corresponding control (dual reporter plus empty TALE-TF vector).

The above findings indicate that the dual reporters are specific and robust (> 200 fold increase), as demonstrated by two sets of vectors. To further examine the sensitivity of this dual reporter, we performed dose–response experiments. As shown in Figure 3a, as low as 1 ng effector DNA induced GFP positive cells (white arrows), and the number of GFP positive cells increased in parallel with the increasing dosages of effector DNA to a maximum of 2.5 ug tested. Consistent with GFP results, there was also a dose-dependent increase in luciferase activity. Specifically, the lowest dose of 1 ng effector DNA input resulted in a significant 15-fold increase compared to the empty-vector DNA control, while the highest dose of 2.5 ug resulted in a marked 9847-fold increase in luciferase activities. These data strongly support the notion that these dual reporters may serve as a robust functional validation system for customized TALE-TFs with both high sensitivity and specificity.

### Monitoring TALE-TF action in real-time

**Figure 4:**
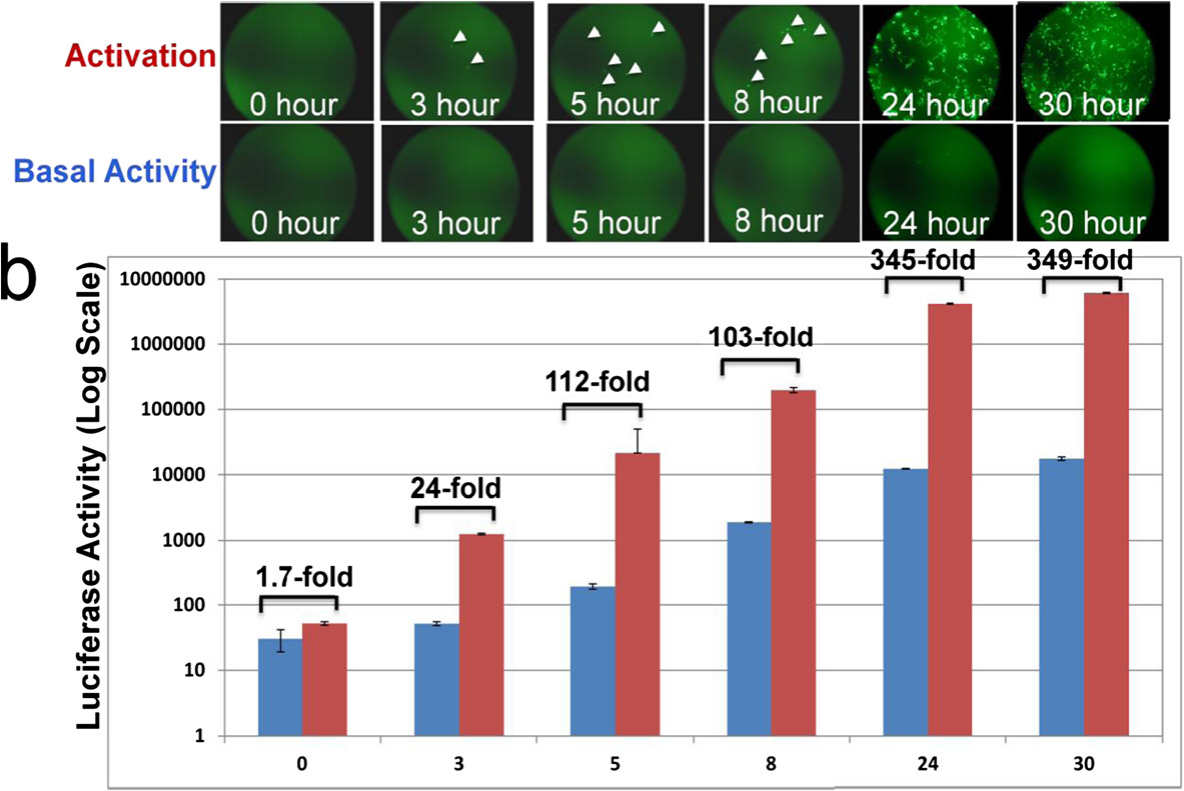
**Real-time monitoring and quantification of TALE in living cells. **Images of TALE-induced GFP reporter expression in HEK293 cells were taken at indicated time-points after co-transfection of Sox2-TALE-TF and its dual reporter. (**b**) Fold induction of luciferase was calculated as relative activity at each time-point compared to its corresponding control (dual reporter plus empty TALE-TF vector). Levels of luciferase activities are expressed as mean ± SD, n = 3.

One important advantage for the use of dual reporters is that GFP allows real-time monitoring in living cells, while luciferase enables easy quantification. To test the ability of GFP as a visual tool to monitor TALE-mediated gene regulation, we conducted time-course studies using our dual reporters. As shown in Figure 4a, as early as 3 hours after transfection, a few weak GFP-positive cells were observed in HEKs co-expression of Sox2-TALE-TF with dual reporters. The number of GFP-positive cells and the intensity increased with time, reached a peak at 24 hours, and then attenuated at 30 hours. Under the same experimental condition, both the number of GFP positive cells and the intensity are much lower in the empty-vector DNA (no TALE-TF) controls (Figure 4a, lower panel), with an apparent time-delay. In agreement with GPF results, there is a corresponding increase in luciferase activity in a time-dependent manner (Figure 4b) These results confirm the specific effects of TALE-TF, as well as the feasibility of real-time monitoring of TALE-TF action by our dual reporter system. Together, these observations provide a strong correlation between the visual GFP assessment and luciferase quantification.

## Conclusion

We have designed and built a dual reporter system for the functional evaluation of TALE-TF. In addition, we have validated specificity and sensitivity of this robust reporter system. Since this dual reporter system allows real-time monitoring and cost-effective quantification, it will add a great value to the community using TALE-TF models to study gene regulation in mammalian cells.

## Methods

### Construction of dual reporters and TALEs

The dual reporter system is configured from the 5’ to 3’ as follows: a transcription stopper sequence, MCS, mCMV, GFP-T2A-Luciferase, followed by a Poly A signal (core sequence). The components of the core sequence were PCR amplified and fused together using gene fusion technology (SBI, Mountain View, CA). The above dual reporter sequences were subsequently cloned into either a regular plasmid with an ampicillin selection marker or a minicircle plasmid with a kanamycin selection marker (SBI, Mountain View). In addition, reporters without mCMV or with a full-length CMV promoter sequences were similarly constructed to serve as negative or positive controls. For TALE transcription activators, we placed the Sox2 gene activator into a lentiviral vector under the control of CMV. This Sox2-TALE-TF is designed to target a 14-mer sequence site located at the proximal promoter of the human Sox2 gene. Three binding repeats of the Sox2 14-mer or three repeats of unrelated 14-mers as negative control were subcloned into the MCS of the above dual reporters.

### Cell culture

HEK cells were maintained in high glucose Dulbecco’s Minmal Essential Medium (DMEM) supplemented with 10% FBS, 2 mM GlutaMax (Life Technologies), penicillin/streptomycin. Cells were incubated at 37°C and 5% CO_2_. At ~80% confluence, cells were washed with PBS and passaged with 0.25% trypsin-EDTA for dissociation.

### Transfection and reporter gene assay

For transfections, HEK cells were seeded on 6-well plates at a density of 1 x 10^5^/well, and a total of 1 ug dual reporter and 2 ug effector DNA (Sox2-TALE-TF or empty vector DNA mock control) with polymer-based transfection reagent (SBI, Mountain view, CA) were added to the media following manufacturer’s instruction. If not stated otherwise, cells were harvested 18 hours after transfection for measuring luciferase activity, using a Luciferase assay kit according to manufacturer’s instructions (Promega, Madison, WI).

### Data collection and presentation

For real-time monitoring, cultured cells were monitored at regular time intervals under a fluorescent microscope. GFP live cell images were taken at indicated time-points using same exposure condition within the group of comparison. For luciferase reporter assay, all data are presented as mean  ±  S.D. (n = 3), unless stated otherwise.

## Abbreviations

TALE: Transcription activator-like effector; GFP: Green fluorescent protein; HTP: High through put; mCMV: Minimal cytomegalovirus promoter; SBS: Sox2-binding site; UBS: Unrelated binding site.

## Competing interests

All authors in this manuscript declare that we have no significant competing financial, professional interests that might have influenced the performance or presentation of the work described in this manuscript.

## Authors’ contributions

BL and CS conceived the study aims and design, contributed to the systematic review and data extraction, performed the analysis and interpreted the results. BL performed the experiments and drafted the manuscript. CS and HJ contributed to the revision of the manuscript. All authors have read and approved the final version of this paper.
